# Supplementary material for: Further Evidence of Neuroprotective Effects of Recombinant Human Erythropoietin and Growth Hormone in Hypoxic Brain Injury in Neonatal Mice
Source: Int J Mol Sci. 2022 Aug 4;23(15):8693. doi: 10.3390/ijms23158693 (PMC9368903; doi:10.3390/ijms23158693)
Supplement: Supplementary file 1 [file ijms-23-08693-s001.zip › ijms-1830651-supplementary.pdf]

Supplementary Table S1. Cerebral gene expression of VEGF receptors in normoxic and hypoxic brains of neonatal mice (P7) treated with vehicle (VT, NaCl 0.9%, i.p.), growth hormone (rhGH, 4000 µg/kg, i.p.), or erythropoietin (rhEPO, 5000 IU/kg, i.p.) compared to non-treated (NT) controls. Gene expression (RT PCR) related to ß-actin mRNA levels was assessed after a regeneration period of 48 h (**A**) or 7 d (**B**); n = 5. There were no statistically significant differences between the groups (two-way ANOVA).

A

	**non-treated**			**vehicle-treated**			**rhGH (4000 µg/kg)**			**rhEPO (5000 IU/kg)**		
**[x10^{-3}]**	**normoxia**	**hypoxia**	P value	**normoxia**	**hypoxia**	P value	**normoxia**	**hypoxia**	P value	**normoxia**	**hypoxia**	P value
**VEGFR-1**	1.0±0.2	0.9±0.2	ns	1.2±0.1	1.4±0.1	ns	1.1±0.1	1.0±0.1	ns	1.3±0.2	1.2±0.2	ns
**VEGFR-2**	4.5±0.8	4.8±0.9	ns	4.3±0.5	6.0±0.6	ns	4.5±0.5	5.8±1.0	ns	5.5±0.5	4.4±0.1	ns

B

	**non-treated**			**vehicle-treated**			**rhGH (4000 µg/kg)**			**rhEPO (5000 IU/kg)**		
**[x10^{-3}]**	**normoxia**	**hypoxia**	P value	**normoxia**	**hypoxia**	P value	**normoxia**	**hypoxia**	P value	**normoxia**	**hypoxia**	P value
**VEGFR-1**	36.9±4.4	37.1±3.6	ns	38.1±3.2	37.0±3.5	ns	32.9±4.2	34.8±2.8	ns	29.9±6.0	24.6±3.5	ns
**VEGFR-2**	14.9±1.1	16.1±0.9	ns	14.3±1.5	16.3±2.1	ns	13.7±0.6	15.3±1.1	ns	13.9±1.3	13.5±0.9	ns

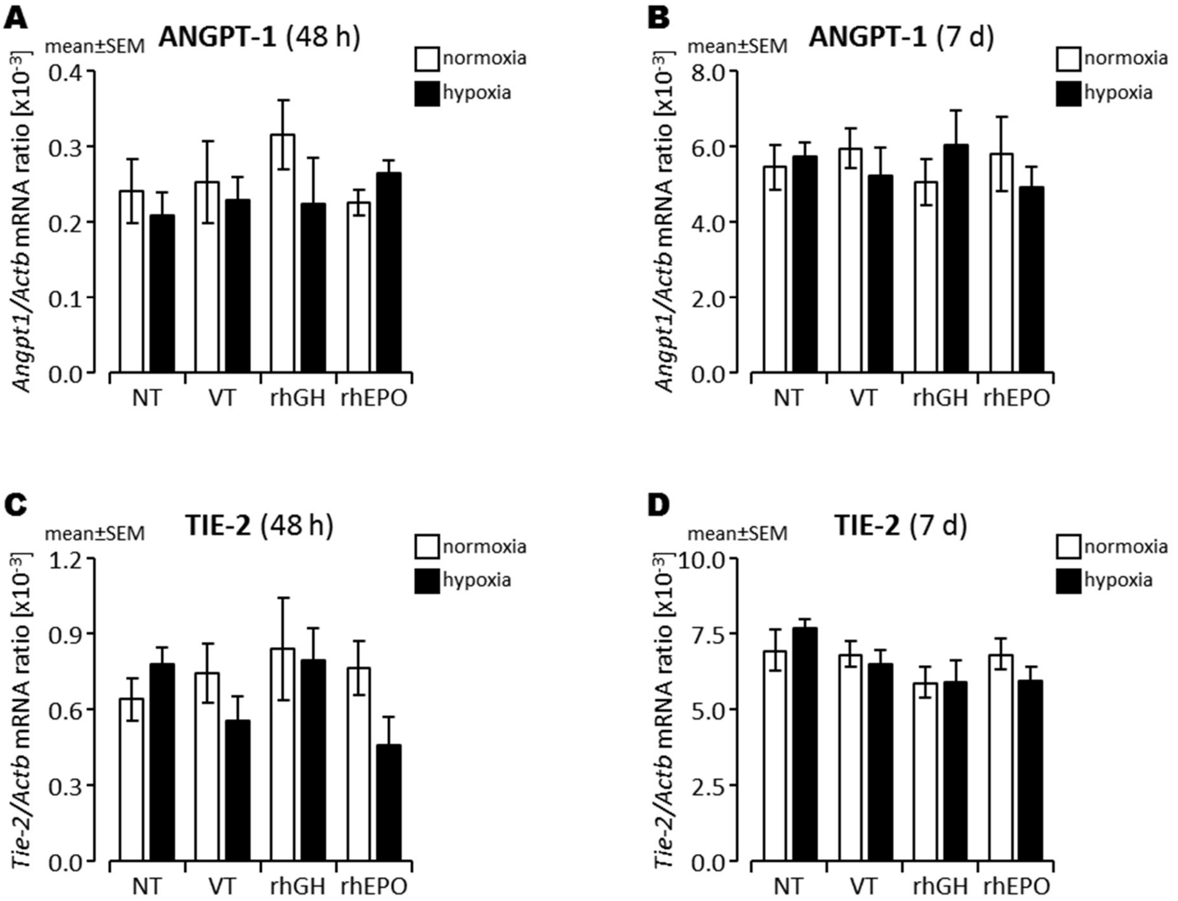

Supplementary Figure S1. Gene expression of ANGPT-1 (**A**, **B**) and TIE-2 (**C**, **D**) in relation to ß-actin mRNA in normoxic and hypoxic mouse brains (P7) treated with vehicle (VT, NaCl 0.9%, i.p.), growth hormone (rhGH, 4000 µg/kg, i.p.), or erythropoietin (rhEPO, 5000 IU/kg, i.p.). NT, non-treated controls. Gene expression (RT PCR) was assessed after a regeneration period of 48 h (**A**, **C**) or 7 d (**B**, **D**); n = 5.

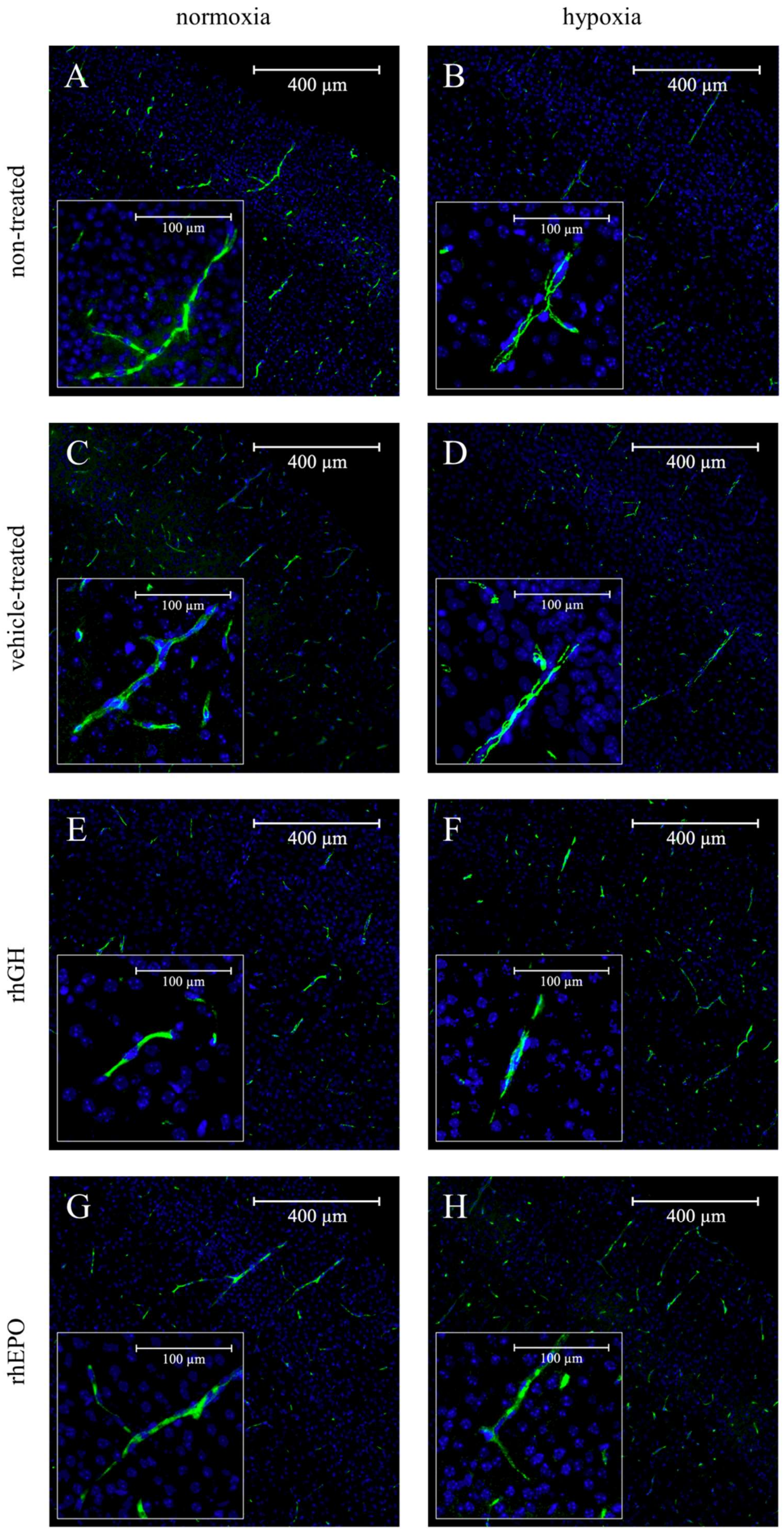
normoxia
hypoxia
non-treated
vehicle-treated
rhGH
rhEPO
A
B
C
D
E
F
G
H
400 µm
100 µm

Supplementary Figure S2. Representative photomicrographs of PECAM-1 protein staining (green) of vascular endothelial cells in hypoxic developing mouse brains with and without rhGH and rhEPO treatment after a 48-h regeneration period in NT, VT, rhGH treated, and rhEPO treated brains in the parietal cortex. Blue, 4′,6-diamidino-2-phenylindole (DAPI) nuclear counterstain.

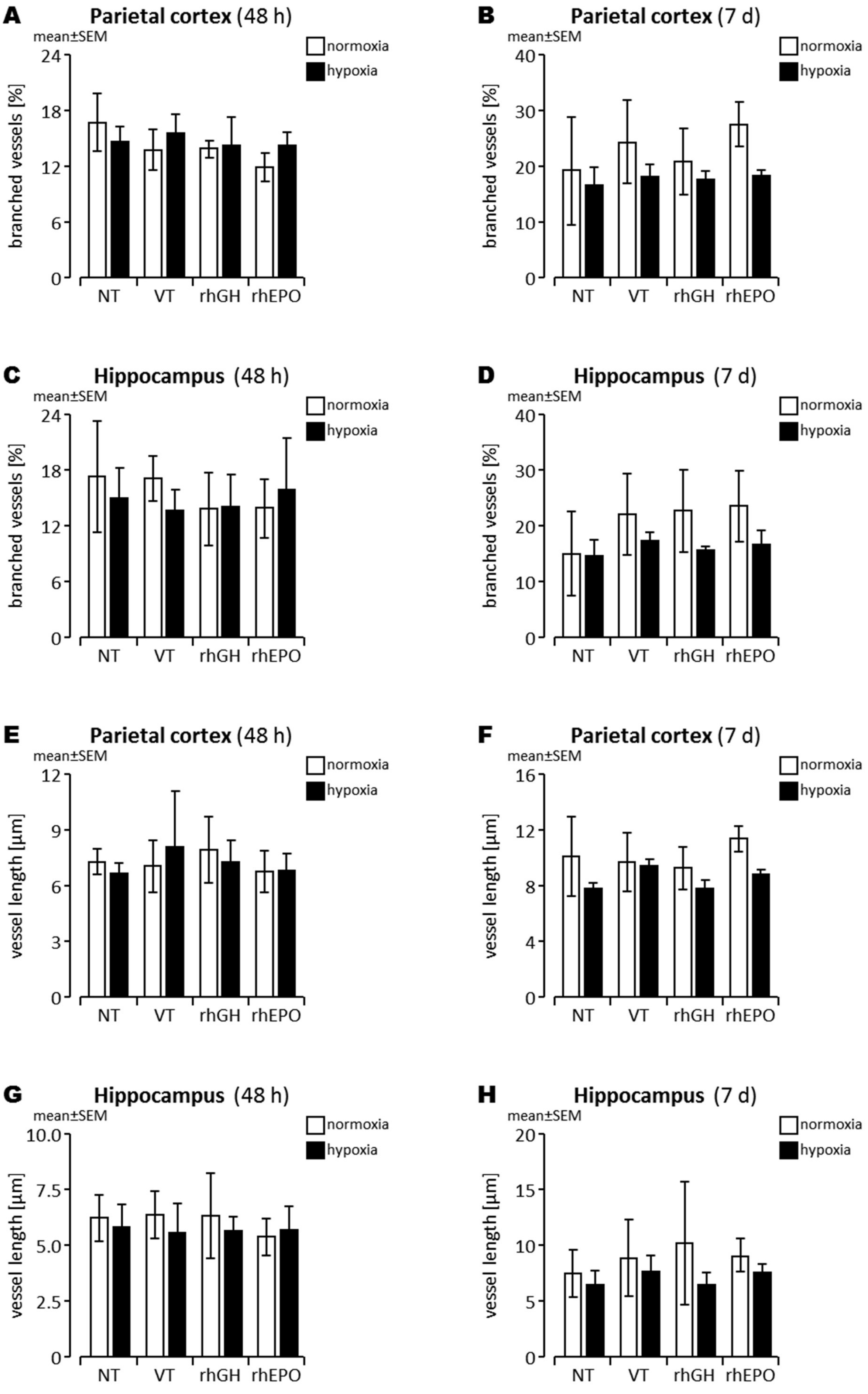
A
Parietal cortex (48 h)
mean±SEM
normoxia
hypoxia
branched vessels [%]
24
18
12
6
0
NT
VT
rhGH
rhEPO
B
Parietal cortex (7 d)
mean±SEM
normoxia
hypoxia
branched vessels [%]
40
30
20
10
0
NT
VT
rhGH
rhEPO
C
Hippocampus (48 h)
mean±SEM
normoxia
hypoxia
branched vessels [%]
24
18
12
6
0
NT
VT
rhGH
rhEPO
D
Hippocampus (7 d)
mean±SEM
normoxia
hypoxia
branched vessels [%]
40
30
20
10
0
NT
VT
rhGH
rhEPO
E
Parietal cortex (48 h)
mean±SEM
normoxia
hypoxia
vessel length [µm]
12
9
6
3
0
NT
VT
rhGH
rhEPO
F
Parietal cortex (7 d)
mean±SEM
normoxia
hypoxia
vessel length [µm]
16
12
8
4
0
NT
VT
rhGH
rhEPO
G
Hippocampus (48 h)
mean±SEM
normoxia
hypoxia
vessel length [µm]
10.0
7.5
5.0
2.5
0
NT
VT
rhGH
rhEPO
H
Hippocampus (7 d)
mean±SEM
normoxia
hypoxia
vessel length [µm]
20
15
10
5
0
NT
VT
rhGH
rhEPO

Supplementary Figure S3. Vascular development in developing mouse brain exposed to acute hypoxia (8% O_2) or normoxia and treated with vehicle (VT, NaCl 0.9%, i.p.), growth hormone (rhGH, 4000 μg/kg, i.p.), or erythropoietin (rhEPO, 5000 IU/kg, i.p.) in relation to non-treated (NT) controls. After a regeneration period of 48 h (**A**, **C**, **E**, **G**) and 7 days (**B**, **D**, **F**, **H**), vessel branching and vessel length in the parietal cortex (**A**, **B**, **E**, **F**) and the hippocampus (**C**, **D**, **G**, **H**) were quantified by PECAM-1 IHC. Data are expressed as mean ± SEM; n = 5 per group, ** $p < 0.01$.

Supplementary Table S2. Gene expression of tight junction proteins (related to PBGD, mean±SEM) in response to rhGH or rhEPO in normoxic and hypoxic brains after a 48-h (**A**) or 7-d period of regeneration (**B**) in comparison to controls; n = 5 per group. There were no statistically significant differences between the groups (two-way ANOVA).

A

	**non-treated**		**vehicle-treated**		**rhGH (4000 µg/kg)**		**rhEPO (5000 IU/kg)**	
	normoxia	hypoxia	normoxia	hypoxia	normoxia	hypoxia	normoxia	hypoxia
**ZO-1**	3.49±0.14	3.74±0.55	3.47±0.49	3.48±0.48	3.64±0.46	4.15±0.40	4.29±0.30	4.10±0.55
**Claudin-1**	0.58±0.03	0.54±0.02	0.48±0.02	0.60±0.02	0.54±0.01	0.55±0.03	0.55±0.01	0.61±0.06
**Claudin-5**	2.17±0.11	1.82±0.07	2.10±0.17	1.96±0.23	2.33±0.07	2.18±0.13	2.13±0.13	1.92±0.14

B

	**non-treated**		**vehicle-treated**		**rhGH (4000 µg/kg)**		**rhEPO (5000 IU/kg)**	
	normoxia	hypoxia	normoxia	hypoxia	normoxia	hypoxia	normoxia	hypoxia
**ZO-1**	13.93±3.02	11.74±3.38	16.62±4.39	8.89±1.40	10.05±1.19	10.19±3.24	9.79±1.46	15.03±5.52
**Claudin-1**	1.00±0.07	0.87±0.16	0.92±0.03	0.77±0.08	0.91±0.09	1.37±0.68	0.97±0.11	0.91±0.11
**Claudin-5**	3.16±0.33	2.63±0.80	3.61±0.49	2.52±0.09	3.01±0.42	3.39±1.49	2.94±0.45	2.50±0.66

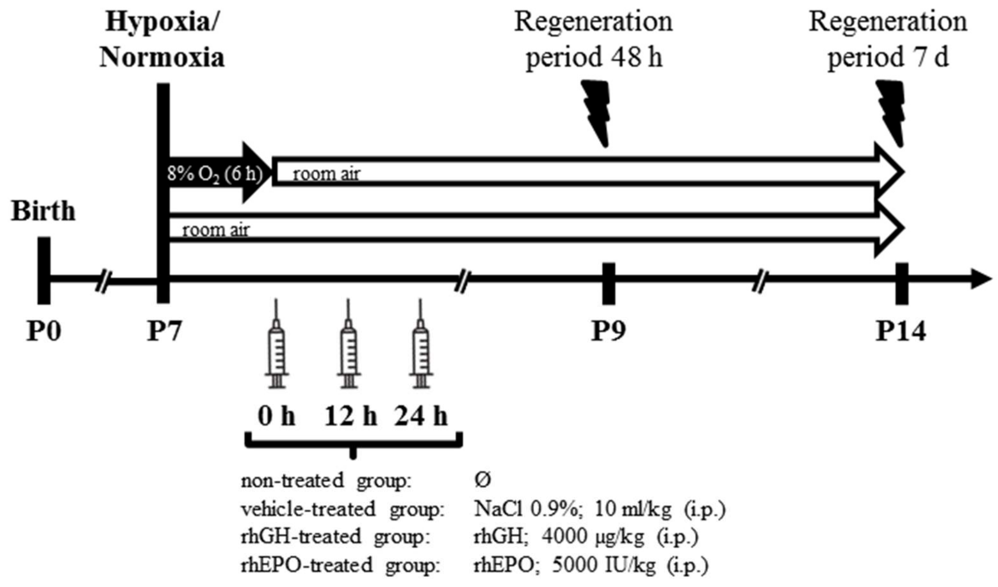

Supplementary Figure S4. Experimental design. P, postnatal day.
